# Supplementary material for: Modeling glioblastoma heterogeneity as a dynamic network of cell states
Source: Mol Syst Biol. 2021 Sep 16;17(9):e10105. doi: 10.15252/msb.202010105 (PMC8444284; doi:10.15252/msb.202010105)
Supplement: Supplementary file 5 — Source Data for Figure 3 [file MSB-17-e10105-s001.zip › Figure3A_sourcedata/GSEA_3065/nosigdb_state4.GseaPreranked.1622718430994/NB_KI_CLUSTER1.html]

Details for gene set NB\_KI\_CLUSTER1[GSEA]

|  || Dataset | state4 |
| Phenotype | NoPhenotypeAvailable |
| Upregulated in class | na\_pos |
| GeneSet | NB\_KI\_CLUSTER1 |
| Enrichment Score (ES) | 0.8672128 |
| Normalized Enrichment Score (NES) | 2.8923645 |
| Nominal p-value | 0.0 |
| FDR q-value | 0.0 |
| FWER p-Value | 0.0 |
Table: GSEA Results Summary

  

Fig 1: Enrichment plot: NB\_KI\_CLUSTER1      
 Profile of the Running ES Score & Positions of GeneSet Members on the Rank Ordered List

  

| PROBE | GENE SYMBOL | GENE\_TITLE | RANK IN GENE LIST | RANK METRIC SCORE | RUNNING ES | CORE ENRICHMENT || 1 | EEF1A1 |  |  | 12 | 0.471 | 0.0762 | Yes |
| 2 | TPT1 |  |  | 58 | 0.292 | 0.1172 | Yes |
| 3 | RPL34 |  |  | 69 | 0.278 | 0.1617 | Yes |
| 4 | RPS27 |  |  | 98 | 0.250 | 0.1987 | Yes |
| 5 | RPL28 |  |  | 148 | 0.223 | 0.2277 | Yes |
| 6 | RPS19 |  |  | 166 | 0.214 | 0.2605 | Yes |
| 7 | RPL39 |  |  | 168 | 0.214 | 0.2959 | Yes |
| 8 | RPL13A |  |  | 171 | 0.214 | 0.3309 | Yes |
| 9 | RPS3A |  |  | 174 | 0.212 | 0.3658 | Yes |
| 10 | RPS15A |  |  | 188 | 0.207 | 0.3980 | Yes |
| 11 | RPS4X |  |  | 198 | 0.204 | 0.4304 | Yes |
| 12 | RPL21 |  |  | 213 | 0.198 | 0.4610 | Yes |
| 13 | RPS8 |  |  | 221 | 0.193 | 0.4920 | Yes |
| 14 | RPL23A |  |  | 235 | 0.190 | 0.5213 | Yes |
| 15 | RPL13 |  |  | 248 | 0.186 | 0.5502 | Yes |
| 16 | RPLP2 |  |  | 257 | 0.184 | 0.5795 | Yes |
| 17 | RPS12 |  |  | 273 | 0.180 | 0.6069 | Yes |
| 18 | RPLP1 |  |  | 279 | 0.179 | 0.6357 | Yes |
| 19 | RPL10 |  |  | 287 | 0.176 | 0.6638 | Yes |
| 20 | RPL3 |  |  | 333 | 0.169 | 0.6846 | Yes |
| 21 | RPS6 |  |  | 343 | 0.166 | 0.7107 | Yes |
| 22 | RPL32 |  |  | 358 | 0.163 | 0.7355 | Yes |
| 23 | RPS29 |  |  | 376 | 0.158 | 0.7590 | Yes |
| 24 | RPS18 |  |  | 377 | 0.158 | 0.7853 | Yes |
| 25 | RPS27A |  |  | 395 | 0.153 | 0.8079 | Yes |
| 26 | RPL41 |  |  | 411 | 0.150 | 0.8303 | Yes |
| 27 | RPL27A |  |  | 442 | 0.145 | 0.8494 | Yes |
| 28 | RPL18A |  |  | 550 | 0.131 | 0.8540 | Yes |
| 29 | RPS25 |  |  | 642 | 0.121 | 0.8595 | Yes |
| 30 | RPS2 |  |  | 711 | 0.113 | 0.8672 | Yes |
| 31 | RPS3 |  |  | 1098 | 0.082 | 0.8189 | No |
| 32 | RPS7 |  |  | 1590 | 0.059 | 0.7498 | No |
Table: GSEA details [plain text format]

  

Fig 2: NB\_KI\_CLUSTER1: Random ES distribution      
 Gene set null distribution of ES for **NB\_KI\_CLUSTER1**
